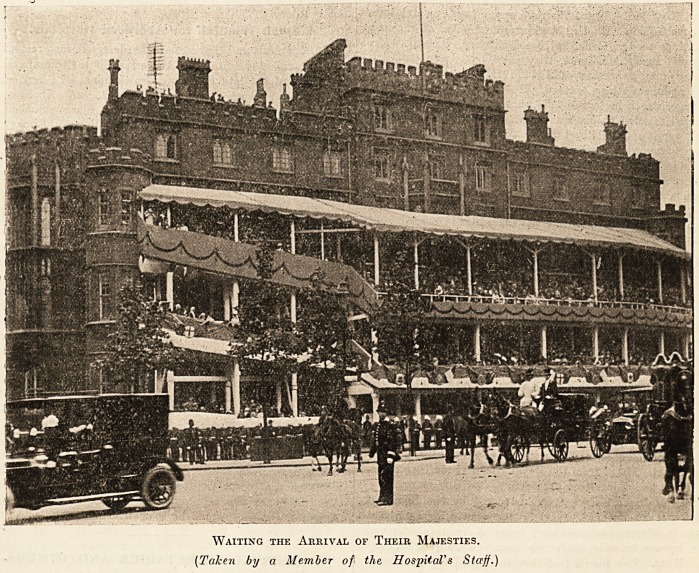# Westminster Hospital Stand

**Published:** 1911-07-01

**Authors:** 


### "WESTMINSTER HOSPITAL STAND."

The photograph below, for which we have to thank Mr. Quennell, the secretary, and a member of the staff, is interesting not merely because it represents, with Westminster Hospital, the finest site in the Metropolis from which to view the Coronation procession, but also because of some idle criticisms that the erection of the stand itself has excited. We are in a position to state that every penny of the amount raised by the leasing of the above seats to the governors and others will be used for the benefit of the patients of the hospital. About 2,000 persons were accommodated on the stand, and we are informed that the posts are still bringing in acknowledgments from the seat-holders for the comfort and attention which were given to them.

The objectors to the stand above referred to tried to justify their criticisms on the ground that the closing of the beds which was made necessary from the noise of the builders was unfair to waiting patients. This objection is worth repeating here as a sample of the ignorance which tries to make a capital out of hospital doings, and its answer may be stated at once. While the wards have been emptied, in consequence of the hammers going on outside, the institution, if not the medical or nursing staff, has been kept busy. The Coronation interval, in fact, has been used thoroughly and promptly to clean the patients' quarters, which, in consequence, have been overhauled in less than half the ordinary time.

Now that the Westminster Hospital is again opening its wards, it opens them with a balance in hand and a scoured interior. Can anyone now deny that the erection of this Coronation stand has fully been justified in the interests of the patients alone? Those who are curious to see what happened at King Edward's Coronation are referred to The Hospital of April 22, 1911, where the part played by this hospital on a similar occasion is described on page 97.

We think that our readers will appreciate this photograph, as an exclusive and authoritative instance of the relations of a London hospital to the great ceremony of last week. The lesson it drives home will not be wasted, we hope, on lay critics of hospitals.

Waiting the Arrival of Their Majesties.
(*Taken by a Member of the Hospital's Staff.*)

## Figures and Tables

**Figure f1:**